# Modeling HIV Vaccine Strategy in Animals

**DOI:** 10.1371/journal.pmed.0020258

**Published:** 2005-07-19

Animal models can play an essential role in guiding preclinical vaccine development, including in studies of preclinical vaccine safety, vaccine toxicity, and vaccine immunogenicity. Appropriate pathogen challenge models can also provide the opportunity to perform preclinical tests of vaccine efficacy. Preclinical tests of HIV vaccine efficacy are usually performed by exposing macaques to simian immunodeficiency virus (SIV), a virus that is closely related to HIV. However, the viral inoculum sizes used to infect macaques with SIV vastly exceed the amounts of HIV that humans are exposed to during a given exposure. Typically animals are exposed to 10–100 times the infectious dose at which 50% of the animals become infected (ID50). These excessive doses may not provide realistic preclinical tests of vaccine efficacy. Indeed, no vaccine has been shown to be effective in preventing infection by SIV (so-called sterilizing immunity) in such high viral inoculum trials. Now, in a paper published in *PLoS Medicine*, Roland Regoes and colleagues speculate that an alternative approach to trials in animals not only can mimic the human patterns of repeated low-dose exposure, but also can remove one concern for animal researchers—the need to use very large numbers of animals in experiments.

What the researchers did was use statistical power analysis to compare a single low-dose challenge design, in which each animal is challenged only once, and a repeated low-dose challenge design, in which each animal is challenged until it is infected or a predetermined maximum number of challenges is reached. The statistical power of an experimental design—a measure of the statistical quality—was assessed by simulating experiments, evaluating them, and then repeating the procedure thousands of times.

What they found was that the experimental design using a single low dose of virus in each animal required unfeasibly large numbers of animals; even for the highest modeled vaccine efficacy of 90% the single low-dose challenge design required more than 20 animals per group to reach a statistical power of 95%. However, when the researchers modeled a protocol of repeatedly challenging the (virtual) animals with a challenge dose of one *ID*_50_, and allowing for a maximum number of 20 challenges of each individual animal, as few as five animals were required to achieve more than 95% of statistical power.

Where do these results leave the design of HIV trials? To begin with, the results should encourage researchers to develop animal models that reflect, to the fullest extent possible, what is known about the natural history and pathogenesis of the disease in humans, rather than designing trials to fit the animal models that are available. The authors have made available the programming script of their analysis so anyone can repeat it; it would be interesting to know whether preclinical trials assessing vaccines or treatments against infections by other pathogens could be usefully modeled in this way as well.

